# Improved tools for efficient mapping of fission yeast genes: identification of microtubule nucleation modifier *mod22-1* as an allele of chromatin- remodelling factor gene *swr1*

**DOI:** 10.1002/yea.1639

**Published:** 2008-12

**Authors:** Andreas Anders, Stephen Watt, Jürg Bähler, Kenneth E Sawin

**Affiliations:** Wellcome Trust Centre for Cell Biology, University of Edinburgh, Swann Building, Mayfield Road, Edinburgh EH9 3JR, UK; Cancer Research UK Fission Yeast Functional Genomics Group, Wellcome Trust Sanger Institute, Hinxton, Cambridge CB10 1HH, UK

**Keywords:** *Schizosaccharomyces pombe*, gene mapping, γ-tubulin, Swr1, H2A.Z

## Abstract

Fission yeast genes identified in genetic screens are usually cloned by transformation of mutants with plasmid libraries. However, for some genes this can be difficult, and positional cloning approaches are required. The mutation *swi5-39* reduces recombination frequency in homozygous crosses and has been used as a tool in mapping gene position (Schmidt, 1993). However, strain construction in *swi5-39*-based mapping is significantly more laborious than is desirable. Here we describe a set of strains designed to make *swi5*-based mapping more efficient and more powerful. The first improvement is the use of a *swi5Δ* strain marked with kanamycin (G418) resistance, which greatly facilitates identification of *swi5* mutants. The second improvement, which follows directly from the first, is the introduction of a large number of auxotrophic markers into mapping strains, increasing the likelihood of finding close linkage between a marker and the mutation of interest. We combine these new mapping strains with a *rec12Δ*-based approach for initial mapping of a mutation to an individual chromosome. Together, the two methods allow an approximate determination of map position in only a small number of crosses. We used these to determine that *mod22-1*, a modifier of microtubule nucleation phenotypes, encodes a truncation allele of Swr1, a chromatin-remodelling factor involved in nucleosomal deposition of H2A.Z histone variant Pht1. Expression microarray analysis of *mod22-1, swr1Δ* and *pht1Δ* cells suggests that the modifier phenotype of *mod22-1* mutants may be due to small changes in expression of one or more genes involved in tubulin function. Copyright © 2009 John Wiley & Sons, Ltd.

## Introduction

The most straightforward method of cloning fission yeast genes is to transform a mutant of interest with a plasmid library and identify transformants with restored wild-type phenotype. This is most often done at the colony level, normally by rescuing the lethality of temperature-sensitive mutants. Alternatively, other ‘macroscopic’ assays are possible, including colour change on phloxin B plates (Zimmerman *et al.*, 2004) or after iodine staining (Ellermeier *et al.*, 2004), or restoration of silencing of *ade6*^+^ on limiting-adenine plates (White, 2008). Rescue of phenotypes after plasmid library transformation can also be assayed microscopically (Chang *et al.*, 1994; K.S., unpublished data), although to date this has generally been put to wider use in budding yeast (see e.g. Harkins *et al.*, 2001).

In some cases, a plasmid-library transformation approach may not be successful. Reasons for this could include toxicity due to plasmid-based overexpression, impracticality in scoring complex (e.g. morphological) or poorly penetrant phenotypes on a large scale, or the absence of a potential rescuing clone from a plasmid library. In such cases, however, it is often possible to use classical genetics to map the position of a mutation. Information about map position can then be used in conjunction with additional methods, such as physical mapping and/or transformation of the mutant strain with cosmids covering the region of interest, in order to clone the gene of interest. Such an approach has been successful, for example, in the cloning of the cell-polarity regulator *tea1*^+^ (Mata and Nurse, 1997), the MAP kinase kinase kinase *win1*^+^ (Samejima *et al.*, 1998) and the spindle pole body protein *cdc11*^+^, part of the septation initiation network (Krapp *et al.*, 2001).

One important advance in determining map position was the introduction of the use of *swi5-39* mutants in the first or second step of mapping (Schmidt, 1993). *swi5* mutants were originally identified on the basis of their reduced mating-type switching in homothallic (*h*^90^) strains (Egel *et al.*, 1984; Gutz and Schmidt, 1985). Subsequently it was shown that *swi5* mutants have a reduced frequency of both intragenic recombination and intergenic recombination, throughout the genome, most likely due to defects in repair or belated repair of DNA double-stranded breaks (De Veaux *et al.*, 1992; Schmidt, 1993; Schmidt *et al.*, 1987, 1989; Young *et al.*, 2004). In spite of this, viability of spores from *swi5*^−^ meioses is comparable to wild-type (Schmidt, 1993; Young *et al.*, 2004). Because genetic distance relative to physical distance is much smaller in *swi5-39* mutants than in wild-type strains (approximately 5–15-fold, depending on the intergenic region), a genetic cross involving *swi5-39* mutants can positively identify linkage between a mutant of interest and even a very distant marker gene. To this end, Schmidt (1993) created several *swi5-39* mapping strains, each of which contained three or four markers on a single chromosome.

In spite of the reduced genetic distance in *swi5-39* mutants and the concomitant benefits for mapping approximate gene position, current methods of *swi5*-based mapping are not ideal. Here we describe significant improvements to this method, using *swi5Δ* strains, and the construction and validation of a generic set of *swi5Δ* mapping strains with many additional auxotrophic markers. We also describe an alternative approach for the initial mapping of a mutation of interest to an individual chromosome, using recombination-deficient *rec12Δ* mutants. We used these methods to determine the map position of a novel mutation, *mod22-1*, which was originally identified as an enhancer of microtubule-nucleation defects when non-essential components of the γ-tubulin complex (γ-TuC) are deleted (Anders *et al.*, 2006). In a further analysis, we found that *mod22-1* is a nonsense allele of the fission yeast homologue of budding yeast chromatin-remodelling factor *SWR1* (Kobor *et al.*, 2004; Krogan *et al.*, 2003; Mizuguchi *et al.*, 2004), and that relatively small changes in gene expression are likely the cause of the *mod22-1* mutant phenotype.

## Materials and methods

Physical positions for various genes were derived from GeneDB at the Wellcome Trust Sanger Institute (http://www.genedb.org/genedb/pombe/), from the genome sequence current on 8 May 2008.

### Yeast strains

Table 1 shows the final strains used for mapping and for our analysis of *mod22/swr1*. A large number of intermediate strains were also constructed (not shown). Apart from standard auxotrophic markers in our laboratory collection, we received marker strains from P. Fantes (University of Edinburgh, UK), G. Smith (Fred Hutchinson Cancer Research Center, Seattle, WA, USA), P. Nurse (Cancer Research UK, London, UK), and C. Hoffman (Boston College, Boston, MA, USA). We received *swi5Δ* (*swi5-201::kanMX6*) and *rec12Δ* (*rec12-168::kanMX6*) from G. Smith (Davis and Smith, 2003; Ellermeier *et al.*, 2004). The *swr1Δ, pht1Δ* and *swc2Δ* deletion strains were obtained from Bioneer (http://pombe.bioneer.co.kr/).

**Table 1:** Fission yeast strains used in this study

Strain	Genotype	Mapping strain number	Source
KS3733	*h*^+^*ura1-161 met5-1 ade3-58 lys2-97 arg3-124 his6-365*	Chr I A1	This study
KS3731	*h*^−^*ura1-161 met5-1 ade3-58 lys2-97 arg3-124 his6-365*	Chr I A2	This study
KS3728	*h*^+^*swi5Δ::kanMX6 ura1-161 met5-1 ade3-58 lys2-97 arg3-124 his6-365*	Chr I A3	This study
KS3729	*h*^−^*swi5Δ::kanMX6 ura1-161 met5-1 ade3-58 lys2-97 arg3-124 his6-365*	Chr I A4	This study
KS3393	*h*^+^*cyh1-7 leu2-120 ade4-31*	Chr I B1	This study
KS3394	*h*^−^*cyh1-7 leu2-120 ade4-31*	Chr I B2	This study
KS3421	*h*^+^*swi5Δ::kanMX6 cyh1-7 leu2-120 ade4-31*	Chr I B3	This study
KS3419	*h*^−^*swi5Δ::kanMX6 cyh1-7 leu2-120 ade4-31*	Chr I B4	This study
KS3402	*h*^+^*ade7-50 his3-D1 can1-1 leu1-32*	Chr II A1	This study
KS3404	*h*^−^*ade7-50 his3-D1 can1-1 leu1-32*	Chr II A2	This study
KS3478	*h*^+^*swi5Δ::kanMX6 ade7-50 his3-D1 can1-1 leu1-32*	Chr II A3	This study
KS3480	*h*^−^*swi5Δ::kanMX6 ade7-50 his3-D1 can1-1 leu1-32*	Chr II A4	This study
KS4251	*h*^+^*leu3-155 lys4-95 arg5-1*	Chr II B1	This study
KS4253	*h*^−^*leu3-155 lys4-95 arg5-1*	Chr II B2	This study
KS4278	*h*^+^*swi5Δ::kanMX6 leu3-155 lys4-95 arg5-1*	Chr II B3	This study
KS4276	*h*^−^*swi5Δ::kanMX6 leu3-155 lys4-95 arg5-1*	Chr II B4	This study
KS4853	*h*^+^*ura4-D18 ade6-M216 arg1-230*	Chr III A1	This study
KS4851	*h*^−^*ura4-D18 ade6-M216 arg1-230*	Chr III A2	This study
KS4855	*h*^+^*swi5Δ::kanMX6 ura4-D18 ade6-M216 arg1-230*	Chr III A3	This study
KS4857	*h*^−^*swi5Δ::kanMX6 ura4-D18 ade6-M216 arg1-230*	Chr III A4	This study
KS3358	*h*^+^*arg1-230 ade5-36*	Chr III B1	This study
KS3356	*h*^−^*arg1-230 ade5-36*	Chr III B2	This study
KS3452	*h*^+^*swi5Δ::kanMX6 arg1-230 ade5-36*	Chr III B3	This study
KS3450	*h*^−^*swi5Δ::kanMX6 arg1-230 ade5-36*	Chr III B4	This study
KS516	*h*^−^*ade6-210 leu1-32 ura4-D18*		Laboratory stock
KS959	*h*^−^*gfh1Δ::kanMX6 ade6-M216 leu1-32 ura4-D18*		Laboratory stock
KS963	*h*^−^*gfh1Δ::kanMX6 mod22-1 ade6-M216 leu1-32 ura4-D18*		Laboratory stock
KS1470	*h*^−^*mod22-1 ade6-M210 leu1-32 ura4-D18*		Laboratory stock
KS3250	*h*^−^*rec12Δ::kanMX6 lys1-37 leu1-32 ade6-M210 his7-366*		G. Smith
KS3320	*h*^+^*swi5Δ::kanMX6*		G. Smith
KS3322	*h*^−^*swi5Δ::kanMX6*		G. Smith
KS3349	*h*^+^*gfh1Δ::hphMX6 mod22-1*		This study
KS3484	*h*^+^*swi5Δ::kanMX6 gfh1Δ::hphMX6 mod22-1*		This study
KS3488	*h*^−^*swi5Δ::kanMX6 gfh1Δ::hphMX6 cyh1-7 leu2-120 ade4-31*		This study
KS3611	*h*^−^*gfh1Δ::hphMX6 cyh1-7 leu2-120 ade4-31*		This study
KS4348	*h*^+^*swr1Δ::kanMX6 ade6-210 leu1-32 ura4-D18*		Bioneer
KS4350	*h*^−^*gfh1Δ::hphMX6 swr1Δ::kanMX6*		This study
KS4355	*h*^+^*pht1Δ::kanMX6 ade6-216 leu1-32 ura4-D18*		Bioneer
KS4362	*h*^−^*swr1ΔC::kanMX6 ade6-210 leu1-32 ura4-D18*		This study
KS4367	*h*^+^*gfh1Δ::hphMX6 pht1Δ::kanMX6 ade6-216 leu1-32*		This study
KS4384	*h*^+^*gfh1Δ::hphMX6 swr1ΔC::kanMX6 ade6-210 leu1-32 ura4-D18*		This study
KS4555	*h*^−^*gfh1Δ::hphMX6 mod22-1 leu1-32:p[leu1*^+^*:nmt1:swr1-Flag-Flag-His]*		This study
KS4645	*h*^+^*swc2Δ::kanMX ade6-216 leu1-32 ura4-D18*		Bioneer
KS4666	*h*^+^*gfh1Δ::hphMX6 swc2Δ::kanMX leu1-32 ura4-D18*		This study

### Media and crosses

Standard fission yeast genetic techniques were used throughout (Moreno *et al.*, 1991). Media were either yeast extract medium (YE5S) or Edinburgh minimal medium (EMM), or EMM plus glutamate (EMMG; EMM using 5 g/l Na glutamate as nitrogen source; also known as PMG). Supplements were used at 150–200 g/l. Canavanine (CAN) was added as hydrochloride from a filter-sterilized stock to autoclaved medium to a final concentration of 100 µg/ml. Cycloheximide (CHX), G418, nourseothricin and Hygromycin B were added to a final concentration of 100 µg/ml. Genetic crosses were carried out at 28 °C for 2–3 days on sporulation agar (SPA), including supplements as required. Tetrad analysis was done using a Singer micromanipulator on YE5S plates. Map distance was determined as described previously (Schmidt, 1993), using Perkins' (1949) formula and also a formula based on maximum-likelihood estimates (Munz *et al.*, 1989).

### Genomic DNA sequencing

Genomic DNA was amplified by yeast colony polymerase chain reaction (PCR), using a blend of *Pwo* and *Taq* polymerase. The resulting 2 kb PCR products were treated with exonuclease I and Antarctic phosphatase (NEB) and directly used in sequencing reactions. Lasergene (DNAStar) was used to assemble and analyse the sequences.

### Truncation of Swr1

Truncation of Swr1 (*swr1ΔC*) was achieved by PCR-based one-step homologous recombination according to Bähler *et al.* (1998). The *kanMX* cassette was amplified by PCR from a plasmid, using a forward oligonucleotide corresponding to 80 bp upstream of the truncation site, but carrying a stop codon at its 3′-end, and a reverse oligonucleotide corresponding to 80 bp downstream of the original stop codon. The amplified fragment was transformed into a wild-type strain and stable integrants were selected. The truncation was confirmed by colony PCR.

### Phenotype assay and microscopy

For morphology experiments, cell shape defects were determined by growing cells on YE5S plates for 2 days, replica-plating to fresh plates, and examining cell shape after 3 h at 32 °C (Snaith and Sawin, 2003). For imaging of cell shape by differential interference contrast (DIC) microscopy, cells were washed off the plates with deionized water and immediately fixed in 3% formaldehyde.

### Phenotype rescue of *gfh1Δ mod22-1* by *swr1*

The *swr1* genomic clone (39/D03) from the ORFeome collection (Matsuyama *et al.*, 2006) was cloned into pDUAL–FFH1c (Matsuyama *et al.*, 2004) to create a *leu1*-integratable plasmid expressing C-terminally-tagged Swr1-2 × FLAG–6His under control of the *nmt1* promoter. The plasmid was linearized with *Not*I and used for transformation of an *Sz. pombe* strain carrying the *mod22-1* mutation in a *gfh1Δ* background. The phenotype of a clone stably expressing Swr1–2 × FLAG–6His was determined in the morphology assay described above (i.e. under repressing conditions for *nmt1*).

### Microarray experiments

RNA isolation was performed as described (Lyne *et al.*, 2003). Cy3 and Cy5 (GE Healthcare) incorporation was carried out using the Invitrogen Superscript direct cDNA labelling system, according to the manufacturer's instructions. Mutant samples were hybridized against wild-type samples on the same array. Two independent biological repeats were carried out, and dye swaps were applied for repeated experiments. Microarrays were scanned using an Axon GenePix 4000B scanner and analysed with GenePix 6.0 software. Quality control and data normalization was carried out as described (Lyne *et al.*, 2003). Results were analysed with GeneSpring GX 7.3 (Agilent). Microarray data have been deposited in ArrayExpress (http://www.ebi.ac.uk/microarray-as/ae/) under Accession No. E-TABM-517. The processed microarray data are also available at: http://www.sanger.ac.uk/PostGenomics/S_pombe

Average fold-changes from two repeated experiments were used to generate lists of differentially expressed genes. The significance of gene list overlaps was calculated using a standard Fisher's exact test, and the *p* values were adjusted with a Bonferroni multiple testing correction. Each of the six overlap diagrams shown in Figure 3 generated three *p* values, from pairwise comparisons. For the three *p* values associated with Figure 3A, ‘> 1.25× down’, all *p* values were < 1 × 10^−59^. For Figure 3A, ‘> 1.5× down’, all *p* values were < 7 × 10^−25^, and for ‘> 2× down’, all were < 4 × 10^−23^. For the three *p* values associated with Figure 3B, ‘> 1.25× up’, all *p* values were < 1 × 10^−06^. For Figure 3B, ‘> 1.5× up’, all *p* values were < 0.0002. For Figure 3B, ‘> 2× up’, *p* values were either 0.03 or not significant.

## Results

The recessive *mod22-1* mutation was originally identified in our laboratory as an enhancer of the microtubule and cell-shape defects seen upon deletion of any one or more of the non-essential γ-tubulin complex (γ-TuC) components Gfh1, Mod21 or Alp16 (Anders *et al.*, 2006; see also Fujita *et al.*, 2002; Venkatram *et al.*, 2004). When grown to stationary phase and then refed with fresh medium, the double mutants *mod22–1 gfh1Δ, mod22–1 mod21Δ* and *mod22–1 alp16Δ* all grow with a curved cell shape, especially during the first cell cycle, as a consequence of a reduced frequency of cytoplasmic microtubule nucleation (Anders *et al.*, 2006). By contrast, the single mutants *gfh1Δ, mod21Δ* and *alp16Δ* have a less pronounced reduction in microtubule nucleation, and thus tend to grow as straighter cells. The *mod22-1* single mutant has nearly no observable morphological phenotype on its own, and this has suggested that *mod22*^+^ may be particularly important for γ-TuC-mediated microtubule nucleation when the large γ-TuC is not fully functional (Anders *et al.*, 2006). We initially attempted to clone *mod22*^+^ by transformation of *mod22–1 gfh1Δ* double mutant cells with a plasmid library, followed by microscopy of individual replica-plated colonies to identify phenotype rescue. However, we recovered only *gfh1*^+^-containing plasmids (P.C.C. Lourenco and K.S., unpublished data). In a further set of experiments we transformed *mod22-1 gfh1Δ mod21Δ cdc25-22* quadruple mutants, which might be rescued only by *mod22*^+^ and not by *gfh1*^+^ or *mod21*^+^ (the *cdc25-22* mutation enhances the curved-cell phenotype at semi-permissive temperatures, facilitating rapid visual screening), but we did not identify any phenotype rescue in nearly 80 000 transformants. This suggested either that *mod22*^+^ may not be properly represented in our plasmid library, or that this method would not work for *mod22*^+^ in principle. Because of the time-intensive nature of microscopic screening, we undertook an alternative approach, involving positional gene mapping.

### *rec12Δ* mapping to determine chromosomal location

A typical first step in positional mapping in fission yeast is to determine whether a gene of interest is located on chromosome I, II or III. Historically this has often been done by using the mutant mating-type allele *mat2-B102* (also known as *mei1-B102)* to construct stable, non-sporulating diploids heterozygous for both the mutation of interest and different auxotrophic markers on each chromosome (Kohli *et al.*, 1977). Haploidization is then induced using drugs such as fluoro-phenylalanine, thiabendazole or methyl benzimidazol-2yl carbamate, generating haploid strains without any prior meiotic crossing-over. Analysis of co-segregation of the mutation of interest with the auxotrophic markers can then reveal on which chromosome the mutation resides (Kohli *et al.*, 1977). We took an alternative approach, using a *rec12Δ::kanMX6* mutant with distinct markers on each chromosome (provided by Gerry Smith, Fred Hutchinson Cancer Research Center). *rec12Δ* mutants are viable but exhibit up to a 1000-fold reduction in recombination in homozygous crosses, reducing the total number of crossovers per meiosis per genome from 40–45 to practically nil (Davis and Smith, 2003; De Veaux *et al.*, 1992), and the *rec12Δ::kanMX6* allele can be easily identified in strain constructions by G418-resistance. *rec12Δ*-based mapping requires that both mating partners in a mapping cross must be *rec12Δ*; thus, depending on the complexity of the circumstances, one should be able to construct strains and map a mutation to an individual chromosome in two or three conventional crosses.

We constructed *rec12Δ gfh1Δ mod22-1 ade6-210 leu1-32 lys1-37* mutants and crossed them to *rec12Δ gfh1Δ* mutants. The resulting progeny showed 96% co-segregation of *mod22-1* with *lys1-37*, and random segregation of *mod22-1* with *ade6-210* and *leu1-32* markers, indicating that *mod22*^+^ is located on chromosome I.

### *swi5Δ* mapping strains for individual chromosomes

For *swi5*-based mapping, both mating partners must be *swi5*^−^ mutants, and therefore mutations of interest must be crossed into *swi5*^−^ backgrounds. However, identification of *swi5-39* mutants is most easily done by iodine staining on sporulation plates, which is easy in homothallic (*h*^90^) strains but not possible in the heterothallic *h*^−^ or *h*^+^ strains that are normally used in crosses. Thus, the presence of *swi5-39* in a strain of interest can be determined only retrospectively, involving further crosses (alternatively, a UV-sensitivity assay can be employed; (Schmidt *et al.*, 1989)). This can make the initial mapping strain construction laborious, especially if additional mutations or markers are to be incorporated, whether to uncover a mutant phenotype (as is necessary with *mod22-1*) or to increase the resolving power of linkage analysis. The *swi5*^+^ gene has been cloned independently by two groups and found to encode a very small (85-amino acid) non-essential protein (Akamatsu *et al.*, 2003; Ellermeier *et al.*, 2004). Interestingly, *swi5Δ::kanMX6* strains have essentially the same phenotype as the original *swi5-39* mutant. Because *swi5Δ::kanMX6* strains can easily be identified by G418-resistance, we used *swi5Δ* strains rather than *swi5-39* mutants to construct strains for mapping crosses.

**Figure 1:**
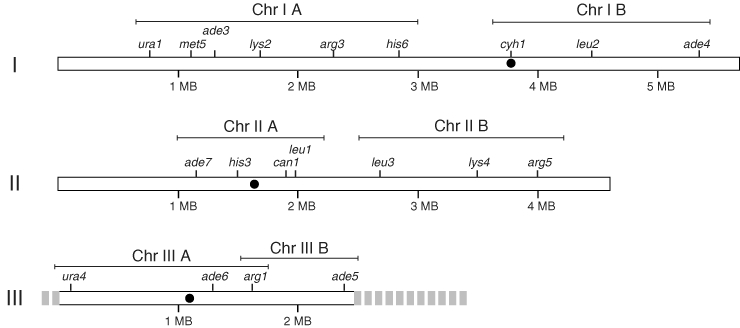
Diagram showing the positions of markers for the six different mapping-strain chromosomes, drawn to scale with the genome sequence. Black circles, centromeres; grey-dashed regions, ribosomal DNA repeats at either end of chromosome III, which can vary in length (Pasero and Marilley, 1993)

We constructed two mapping strains for chromosome I, with six and three markers, respectively, in both *swi5Δ* and *swi5*^+^ backgrounds (Figure 1). We chose easily-scored auxotrophies (Egel, 2004; Kohli *et al.*, 1977; Munz *et al.*, 1989) and in some cases also optimized media conditions for easy screening (Table 2). A cross using our mapping strain for the short arm of chromosome I (chromosome IB) revealed close linkage between *mod22* and *ade4* by tetrad analysis in a *swi5Δ* × *swi5Δ* cross (Table 3). A further cross using the corresponding mapping strain in a *swi5*^+^× *swi5*^+^ context continued to show linkage between *mod22* and *ade4*, with a genetic distance of approximately 8 cM (Table 4). Additional work on the identification and characterization of *mod22*^+^ is presented below.

**Table 2:** Mapping strain markers

Chromosome	Screening medium	Physical position on chromosome^a^ (bp)	Physical distance to next marker gene^a^ (bp)	Genetic distance^a^ (cM)
**I**				
*ura1-161*	EMMG − U	739 300	403 815	50
*met5-1*^b^	EMMG − M	1 143 115	186 147	52
*ade3-58*	EMMG − A	1 329 262	346 468	76
*lys2-97*	EMMG − K^c^	1 675 730	598 282	48
*arg3-124*	EMMG − R	2 274 012	558 784	122
*his6-365*	EMMG − H	2 832 796	937 204^d^	250
*cyh1-7*	YE5S + CHX	3 770 000^d^	670 096^d^	118
*leu2-120*	EMMG − L	4 440 096	924 264	170
*ade4-31*	EMMG − A	5 364 360	N/A	N/A
**II**				
*ade7-50*	EMMG − A	1 157 118	331 093	64
*his3-D1*	EMMG − H	1 488 211	401 953^f^	92
*can1-1*	EMM + CAN^e^	1 890 164^f^	84 324^f^	32
*leu1-32*	EMMG − L	1 974 488	714 104	100
*leu3-155*	EMMG − L	2 688 592	817 459	140
*lys4-95*	EMMG − K^c^	3 506 051	588 597	188
*arg5-1*	EMMG − R	4 094 648	N/A	N/A
**III**				
*ura4-D18*	EMMG − U	115 781	1 200 556	260
*ade6-M210*	EMMG − A	1 316 337	296 987	100
*arg1-230*	EMMG − R	1 613 324	799 041	144
*ade5-36*	EMMG − A	2 412 365	N/A	N/A

a Physical positions are from GeneDB at the Wellcome Trust Sanger Institute (http://www.genedb.org/genedb/pombe/) on 8 May 2008. Genetic distances were estimated using the map of Munz *et al.* (1989).

b *met5* = *met9*.

c Use sodium glutamate as nitrogen source. Ammonium chloride must not be used, as in our hands Ura- Ade- Arg- Lys^+^ strains and Ura^−^Met^−^His^−^Lys^+^ strains failed to grow on ammonium chloride-based medium lacking lysine. There reasons for this are not yet clear.

d Exact position of *cyh1* is unknown and therefore estimated from position of centromere, which is very closely linked.

e Use ammonium chloride as nitrogen source. Sodium glutamate can be used as well, but ammonium chloride gives better results.

f Exact position of *can1* is unknown and therefore estimated from position of *spo14*, which is very closely linked.

N/A, not applicable.

**Table 3:** Mapping of test genes by tetrad analysis

				*swi5*^+^× *swi5*^+^						*swi5Δ* × *swi5Δ*					
Mapping strain	Test gene A	Marker B	Distance A–B (kb)	PD	NPD	T	N	*d*_p_^a^ (cM)	*d*_l_^a^ (cM)	PD	NPD	T	N	*d*_p_^a^ (cM)	*d*_l_^a^ (cM)
Chr I A	*mod21*	*ura1*	521	14	1	29	44	40	61	24	0	0	24	0	0
	*mod21*	*met5*	925	13	4	27	44	58	80	22	0	2	24	4.2	4.4
	*mod21*	*ade3*	1111	11	11	22	44	ND	ND	20	1	3	24	19	12
	*mod21*	*lys2*	1457	11	7	26	44	ND	ND	19	1	4	24	21	14
	*mod21*	*arg3*	2055	11	6	25	44	ND	ND	19	1	4	24	21	14
	*mod2*	*his6*	2614	8	3	23	44	ND	ND	18	1	5	24	23	17
Chr I B	*mod22*	*cyh1*	1507^b^,^c^							18	2	27	47	42	54
	*mod22*	*leu2*	838^b^							30	0	17	47	18	22
	*mod22*	*ade4*	86^b^							45	1	1	47	7.5	3.3
Chr II A	*alp6*	*ade7*	756	8	9	44	61	ND	ND	16	0	12	28	21	28
	*alp6*	*his3*	1087	10	11	40	61	ND	ND	11	1	16	28	39	51
	*alp6*	*can1*	1489^c^	11	14	36	61	ND	ND	9	3	16	28	ND	ND
	*alp6*	*leu1*	1574	10	13	38	61	ND	ND	11	4	13	28	ND	ND
Chr III A	*alp16*	*ura4*	317	12	8	43	63	ND	ND	43	0	11	54	10.2	11.4
	*alp16*	*ade6*	884	12	9	42	63	ND	ND	41	1	12	54	17	23
	*alp16*	*arg1*	1181	17	4	42	63	52	79	33	3	18	54	33	29
Chr III B	*cdc11*	*arg1*	436	13	4	39	56	56	91	35	0	11	46	12	14
	*cdc11*	*ade5*	363	13	8	35	56	ND	ND	43	0	3	46	3.3	3.4

a Map distance *d*_p_ is based on Perkins' formula, and map distance *d*_l_ is derived from maximum likelihood estimate (see Materials and methods), as distances based on Perkins' formula are likely to be underestimates when NPD > 5% of total tetrads. ND, not determined, because data do not suggest linkage.

b Distance determined retrospectively, after identification of *mod22* as *swr1*.

c Estimated distance; see Table 2.

PD, parental ditype; NPD, non-parental ditype; T, tetratype. Our criterion for linkage in tetrad analysis is PD > NPD, with statistical significance *p* < 0.05 by χ^2^ test. A more stringent criterion would be *p* < 0.01 (Kohli *et al.*, 1977).

**Table 4:** Mapping of test genes by random spore analysis

Mapping strain	*swi5* background	Test gene (A)	Marker (B)	Physical distance (A to B; kb)	Recombinants (%)	*n*	χ^2^	*p*
Chr I B	*swi5*^+^	*mod22*	*cyh1*	1507^a^, ^b^	49	120	0.033	0.8551
		*mod22*	*leu2*	838^a^	54	120	0.833	0.3613
		*mod22*	*ade4*	86^a^	8	120	83.333	< 0.0001
Chr II B	*swi5+*	*tea2*	*leu3*	1205	47	280	0.914	0.3390
		*tea2*	*lys4*	387	44	280	3.657	0.0558
		*tea2*	*arg5*	201	25	280	68.014	< 0.0001
Chr II B	*swi5Δ*	*tea2*	*leu3*	1205	13	560	306	< 0.0001
		*tea2*	*lys4*	387	7	560	414	< 0.0001
		*tea2*	*arg5*	201	4	560	475	< 0.0001

a Distance determined retrospectively, after identification of *mod22* as *swr1*.

b Estimated distance; see Table 2.

We validated the *swi5Δ* mapping strain for the long arm of chromosome I (chromosome I A) by tetrad analysis of a cross with a *swi5Δ mod21Δ* strain, in which a non-essential component of the fission yeast γ-tubulin complex is deleted (Anders *et al.*, 2006) (Table 3). In this *swi5Δ* × *swi5Δ* cross, *mod21* showed very close linkage to *ura1*, as well as linkage to other markers, as expected. By contrast, in a comparable *swi5*^+^× *swi5*^+^ cross, only weak linkage of *mod21* to *ura1* was observed, with essentially no linkage to other markers.

The potential general usefulness of this method led us to develop similar mapping strains for chromosomes II and III, in both *swi5Δ* and *swi5*^+^ backgrounds (Figure 1, Table 2). We constructed two strains for chromosome II with seven markers in total, and two strains for chromosome III with four markers in total (see Materials and methods). We validated these strains in a similar manner to the chromosome I strains, by ‘mapping’ the position of known genes involved in cytoskeletal function (Browning *et al.*, 2000; Fujita *et al.*, 2002; Krapp *et al.*, 2001; Tomlin *et al.*, 2002; Vardy and Toda, 2000). All of the mapping strains behaved as desired (Tables 3, 4), revealing linkage between the test gene and at least one auxotrophic marker in all *swi5Δ* × *swi5Δ* crosses, but only rarely in *swi5*^+^× *swi5*^+^ crosses.

### Identification of *mod22* as *swr1*

As described above, with both *swi5Δ* and *swi5*^+^ strains we found close linkage of *mod22* to *ade4* on chromosome I, and additional crosses indicated that *mod22* was approximately 4–8 cM away from *ade4*, towards the centromere.

**Figure 2:**
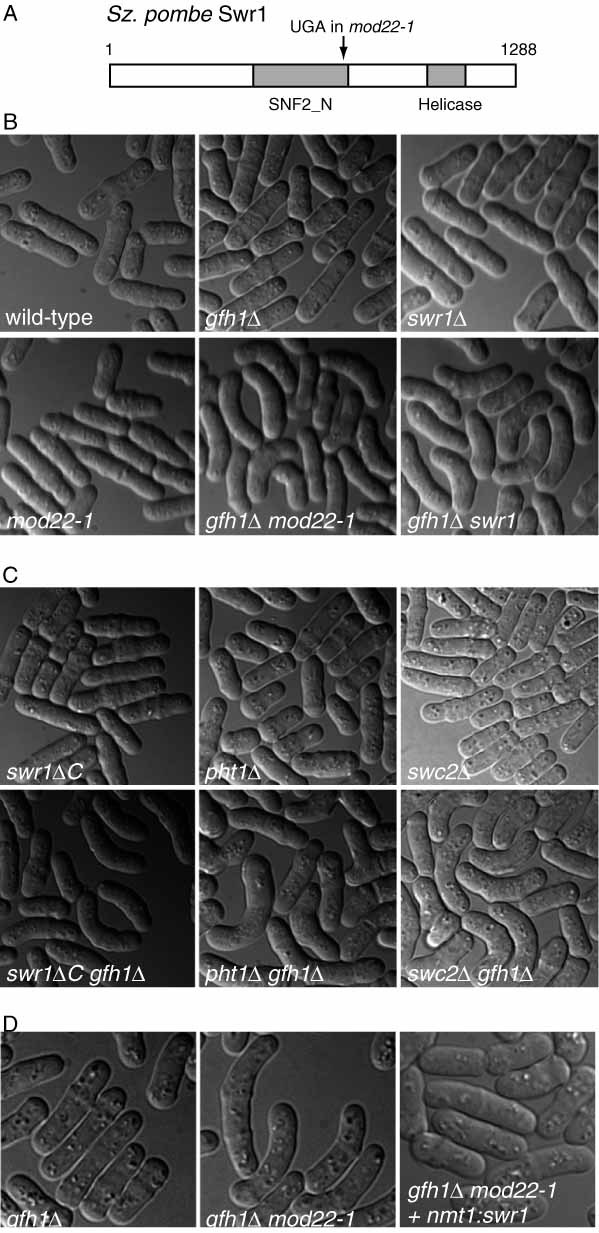
Allelism of *mod22* and *swr1*, a gene encoding a chromatin-remodelling factor. (A) Schematic of fission yeast Swr1 protein, showing position of the nonsense mutation in *mod22-1*. Shaded areas indicate two regions of high identity (>75%) to budding yeast Swr1p. (B) Cell-shape phenotypes in the indicated mutants, showing synthetic effect of combining either *mod22-1* or *swr1Δ* with *gfh1Δ*. (C) Cell-shape phenotypes in the indicated mutants, showing that *swr1ΔC* (a *de novo*-created truncation-equivalent of *mod22-1*), *pht1Δ* (deletion of fission yeast H2A.Z) and *swc2Δ* (deletion of a component of the SWR1–SWR-C complex) all have similar phenotypes to *mod22-1* and *swr1Δ*. D. Rescue of the cell-shape phenotype of *gfh1Δ mod22-1* double mutant by plasmid-based expression of wild-type Swr1

Attempts to rescue the curved-cell phenotype of *mod22-1 gfh1Δ mod21Δ cdc25-22* mutants with cosmids from this region were not successful (data not shown). We therefore directly sequenced a large region of genomic DNA from *mod22-1* mutants, as well as from wild-type control cells. In a 92 kb interval from ORFs SPAC4D7.02c to SPAPJ760.02c, our experimentally determined wild-type sequence showed a 100% match to the published genome sequence (Wood *et al.*, 2002), and we found only a single nucleotide change in *mod22-1* mutants relative to wild-type cells. This was in the uncharacterized ORF SPAC11E3.01c (also known as SPAC2H10.03c), which encodes a 1288 amino acid protein that is most closely related to the budding yeast *SWI2/SNF2* family chromatin-remodelling factor Swr1p (43% identity; Kobor *et al.*, 2004; Krogan *et al.*, 2003; Mizuguchi *et al.*, 2004). We will designate the fission yeast gene as *swr1*^+^ and its protein product as Swr1. The identified mutation is a C to T transition at nucleotide 2209, converting a CGA (Arg) codon to a TGA (stop) codon and truncating the predicted Swr1 protein at about 60% of its predicted length (Figure 2A).

To confirm that *mod22-1* is allelic to *swr1*^+^, we created a *gfh1Δ swr1Δ* double mutant and found that it displayed a curved-cell phenotype, while neither single mutant did (Figure 2B). In addition, using PCR-based gene targeting we created a truncation mutant, *swr1ΔC*, to mimic the nonsense mutation present in *mod22-1*. This, too, led to curved cells in, and only in, *gfh1Δ* backgrounds (Figure 2C). Finally, we transformed the *mod22-1 gfh1Δ* mutant with an integrating plasmid containing *swr1*^+^ (with *nmt1* promoter, repressed) and were able to partially rescue the curved-cell phenotype of the mutant (Figure 2D). These three lines of evidence confirm that *mod22-1* is a nonsense allele of *swr1*.

A large-scale localization screen has shown the fission yeast Swr1 is a nuclear protein (Matsuyama *et al.*, 2006). In budding yeast, Swr1p functions primarily as part of a multi-protein SWR1–SWR-C complex that regulates gene expression by facilitating exchange of variant histone H2A.Z Htz1p into chromatin (Kobor *et al.*, 2004; Krogan *et al.*, 2003; Mizuguchi *et al.*, 2004). This suggested that fission yeast Swr1 may exert its normal function via a similar mechanism, involving Pht1, the fission yeast homologue of Htz1p (Carr *et al.*, 1994). We found that *pht1Δ gfh1Δ* double mutant cells also displayed a curved-cell phenotype, as well as a low frequency of branched cells (Figure 2C). The simplest interpretation of these results is that the phenotypic effects of *mod22-1* and *swr1* mutations are mediated via Pht1 and, more generally, that these effects may be indirect consequences of altered gene expression in the mutants. Consistent with this, when we combined *gfh1Δ* with a deletion of *swc2*, a different component of the SWR1–SWR-C complex (Kobor *et al.*, 2004; Krogan *et al.*, 2003; Mizuguchi *et al.*, 2004), we also obtained a curved-cell phenotype (Figure 2C).

Previous analysis has suggested that *mod22*^+^ is particularly important for microtubule nucleation when the non-essential or ‘non-core’ components of the fission yeast γ-tubulin complex are deleted, leaving behind only the essential core γ-tubulin small complex (γ-TuSC), which contains γ-tubulin (Gtb1) as well as Alp4 and Alp6, the fission yeast homologues of human GCP2 and GCP3 (Anders *et al.*, 2006). To determine whether we could identify one or more critical target genes whose altered expression might be responsible for the *mod22-1*/*swr1Δ* curved-cell phenotype, we performed a transcription microarray analysis of *mod22-1, swr1Δ* and *pht1Δ* mutants. Transcriptome analysis in budding yeast has revealed small but statistically significant changes in the expression of a large number of genes in *swr1Δ* and *htz1Δ* mutants, with considerable overlap between the two mutants, although very few genes show more than even a two-fold change in expression in both mutants (Kobor *et al.*, 2004; Krogan *et al.*, 2003; Mizuguchi *et al.*, 2004). This may be a general feature for mutants affecting chromatin, as similarly small global effects in gene expression were observed in fission yeast mutants affecting gene silencing and RNAi pathways (Hansen *et al.*, 2005). Because of these previous observations, we took a non-conservative approach to analysing our microarray data, in order to include as many potential candidate target genes as possible (see Materials and methods).

**Figure 3:**
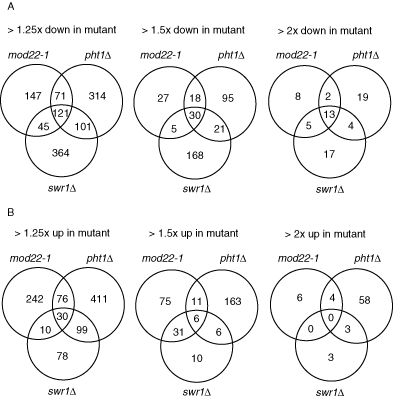
Venn diagrams showing numbers of genes changed in expression in *mod22-1, swr1Δ* and *pht1Δ* mutants, either unique to each mutant, common to pairs of mutants or common to all three mutants. (A) Genes with reduced expression in the mutants. (B) Genes with increased expression in the mutants

We identified 121 genes (out of approximately 5000 in fission yeast) whose average expression was reduced more than 1.25-fold in all three mutants relative to wild-type cells (Figure 3A; see also Supporting information, Table S3, and Materials and methods for statistical significance). Smaller numbers of genes showed increased expression in the mutants (Figure 3B; see also Supporting information, Table S3). Only 13 genes were reduced in expression more than two-fold, and none of these has any known functional link to the microtubule cytoskeleton (Table S3). A possible exception is the gene encoding the CENP-C centromere protein Cnp3 (SPBC1861.01c; SPBC56F2.13), but this is involved in intranuclear mitotic spindle function rather than cytoplasmic microtubule function (Holland *et al.*, 2005).

Interestingly, among the genes reduced more than 1.25-fold in all mutants was the gene encoding the γ-TuSC subunit Alp6, which was reduced in expression an average of 33%, 22% and 38% in *mod22-1, swr1Δ* and *pht1Δ* mutants, respectively. In addition, the gene for ADP-ribosylation factor-like protein Alp41, which is important for the function of tubulin-folding co-factor proteins (Bhamidipati *et al.*, 2000; Radcliffe *et al.*, 2000), was reduced in expression an average of 50%, 42% and 39%, respectively, in the same mutants. A gene encoding a putative tubulin–tyrosine ligase-like protein (SPAC12B10.04) was also reduced in expression an average of 42%, 37% and 58%, respectively, in *mod22-1, swr1Δ* and *pht1Δ* mutants, but the relevance of this remains unclear, as to date there is no evidence for actual tubulin–tyrosine ligase activity in yeast (Badin-Larcon *et al.*, 2004). No other significant changes in gene expression for other γ-TuC proteins, or for tubulin itself, were observed. Overall, these results suggest that the morphological phenotype of *mod22-1, swr1Δ* and *pht1Δ* mutants may be due to small changes in expression of one or more genes, including *alp6* and *alp41*, possibly in conjunction with other genes indirectly affecting microtubule nucleation and/or dynamics.

## Discussion

Here we have described general methods and *swi5Δ* strains that should considerably improve the efficiency of mapping gene position in fission yeast. Using these methods, we were able to show that the enhancement of microtubule nucleation defects by *mod22-1* mutants is due to a premature stop codon in the chromatin-remodelling factor Swr1. Our evidence further suggests that the role of *mod22/swr1* in microtubule nucleation is likely an indirect effect of changes in gene expression. This is not surprising, given the global importance of *SWI2/SNF2* family proteins in regulating chromatin structure in general (Kobor *et al.*, 2004; Krogan *et al.*, 2003; Mizuguchi *et al.*, 2004).

It is much easier to introduce mutations into *swi5Δ* strains than into *swi5-39* strains, and our new strains provide up to nine markers per chromosome, giving higher resolution in the earliest steps of mapping compared to the original strains introduced by Schmidt (1993). With the *rec12Δ* and *swi5Δ* mapping strains, a novel mutation can be mapped to an individual chromosome and further mapped within a given chromosome in a relatively small number of crosses, including both strain construction and the actual mapping crosses. The large number of markers in our mapping strains should make it possible to infer not only the approximate position of a mutation of interest in relation to known markers, but also gene order, as is normally done in ‘three-factor’ crosses, although we did not exploit this directly in the present work. We expect that these strains will provide advantages over previous methods whenever positional mapping is required.

For our mapping strains, we constructed two strains per chromosome. To obtain the most information from the smallest number of crosses, one would like to have as many markers as possible in a single cross, as this not only improves the likelihood of finding linkage but also allows a more accurate determination of approximate map position. However, there are practical reasons against trying to introduce too many markers into a single strain, and against making a single mapping strain for a given chromosome. First, the frequency of obtaining a desired multiple-mutant strain in heterozygous crosses is halved with each additional marker introduced. Second, most chromosomes contain several different auxotrophic markers for the same nutrient (e.g. *ade2, ade3* and *ade4* on chromosome I), and in some cases use of more than one of these may be beneficial. This is easily achieved by putting two markers in different mapping strains. Third, it is likely that too many auxotrophies or specific combinations of auxotrophies in a single strain may compromise growth (Kohli *et al.*, 1977). Indeed, during strain construction we found some unexpected incompatibilities, such as the failure of *his2 leu3 lys4 arg5* quadruple mutants to grow on media individually lacking either methionine, uracil or adenine (data not shown), which forced a change in our strategy for construction of one of the mapping strains for chromosome II.

In constructing general-use *swi5Δ* mapping strains, we used easily-scored markers, almost all of which are auxotrophies, in order for the strains to have a wide application. We avoided using temperature-sensitive alleles, in case the mutation of interest to be mapped was also temperature-sensitive, and we avoided including alleles marked with antibiotic resistance (e.g. nourseothricin or hygromycin; *swi5Δ* is already marked with G418 resistance) because some synthetic phenotypes may require the introduction of such markers for phenotype detection, as with our *gfh1Δ mod22-1* double mutant. However, in some cases, specific additional markers could be included in the mapping strains to provide better resolution, e.g. near the end of the short arm of chromosome II and in chromosome III, which contains a disproportionately small number of known auxotrophies. Further rational modification of the *swi5Δ* mapping strains should be straightforward.

Is it better to use random spore or tetrad analysis with the *swi5Δ* mapping strains? Depending on sample size, tetrad analysis can confidently identify linkage at 50 cM and beyond (with less accuracy in determining actual genetic distance), so in principle it will have an advantage over random spore analysis (Kohli *et al.*, 1977). In essentially all of our *swi5Δ* × *swi5Δ* test crosses, linkage to a marker would most likely have been identified by random spore analysis, based on marker-to-marker distances (see Supporting information, Tables S1 and S2). However, we note that the fold-reduction in recombination in *swi5*^−^× *swi5*^−^ crosses varies across the genome (Schmidt, 1993; also our own data). Therefore, if a mutation of interest were present in a large marker-free interval (e.g. between *ade7* and the telomere on chromosome II; see Figure 1) and recombination were not maximally reduced in this region, it is likely that tetrad analysis would be beneficial.

It is worth pointing out how *swi5Δ* mapping could be used in the context of recent technical advances and genomic resources in fission yeast and beyond. Subsequent to *swi5Δ* mapping, further fine-mapping of a mutation of interest is required before other methods can be used for gene identification (e.g. transforming mutants with phage clones, cosmid clones or ORFeome clones; Matsuyama *et al.*, 2006). For this next step, the *swi5*^+^ mapping strains could be used, or *swi5Δ* strains crossed to the mutant of interest in a *swi5*^+^ background, but in most cases one is unlikely to find linkage to markers. Further fine-mapping could take advantage of non-essential gene deletions from the fission yeast gene deletion set (http://pombe.bioneer.co.kr/). As each deletion is marked with G418 resistance, it would be straightforward to cross a mutation of interest with an evenly-spaced set of deletions.

With the increasing availability of massively parallel sequencing technologies (e.g. Illumina or 454 sequencing), one can identify mutations by sequencing an entire mutant genome at a reasonable price. However, a mutagenized genome from a mutant hunt is likely to contain several mutations, most of which are not of interest. In this case, *swi5Δ* mapping would allow one to focus attention on the region where the mutation of interest is known to reside. In the future, it is also possible that single-nucleotide polymorphisms (SNPs) among different fission yeast strains (e.g. natural geographical isolates vs. laboratory strains; Patch and Aves, 2007) could be used to help map gene position, in conjunction with massively parallel sequencing. Here again *swi5Δ* mapping could be of value, by providing a genomic region of interest for high-resolution SNP-based mapping, and thus reducing the amount of unnecessary screening.

## Supporting Information

Table S1. Distance between markers, from tetrad analysis

Table S2. Distance between markers, from random spore analysis

Table S3. Lists of up- and downregulated genes common to mod22-1, swr1Δ and pht1Δ mutants
