# Supplementary material for: Improved tools for efficient mapping of fission yeast genes: identification of microtubule nucleation modifier mod22-1 as an allele of chromatin- remodelling factor gene swr1
Source: Yeast. 2008 Dec;25(12):913–25. doi: 10.1002/yea.1639 (PMC2964509; doi:10.1002/yea.1639)
Supplement: Supplementary file 1 [file yea0025-0913-SD1.doc]

**Table S1.** Distances between markers, from tetrad analysis

|  |  |  |  |  | ***swi5+* x *swi5+*** | | | | | |  | ***swi5∆* x *swi5∆*** | | | | | |
| --- | --- | --- | --- | --- | --- | --- | --- | --- | --- | --- | --- | --- | --- | --- | --- | --- | --- |
| Mapping strain | Marker  A | Marker  B | Distance  A-B (kb) |  | PD | NPD | T | N | d_p_^a^  (cM) | d_l_^a^  (cM) |  | PD | NPD | T | N | d_p_^a^  (cM) | d_l_^a^  (cM) |
| Chr I A | *ura1* | *met5* | 404 |  | 25 | 0 | 19 | 44 | 22 | 28 |  | 22 | 0 | 2 | 24 | 4.2 | 4.4 |
|  | *met5* | *ade3* | 186 |  | 23 | 4 | 17 | 44 | 47 | 48 |  | 21 | 0 | 3 | 24 | 6.3 | 6.7 |
|  | *ade3* | *lys2* | 346 |  | 18 | 7 | 19 | 44 | 69 | 69 |  | 20 | 0 | 4 | 24 | 8.3 | 9.1 |
|  | *lys2* | *arg3* | 598 |  | 17 | 4 | 23 | 44 | 53 | 61 |  | 24 | 0 | 0 | 24 | 0 | 0 |
|  | *arg3* | *his6* | 559 |  | 20 | 2 | 22 | 44 | 39 | 45 |  | 23 | 0 | 1 | 24 | 2.1 | 2.1 |
|  |  |  |  |  |  |  |  |  |  |  |  |  |  |  |  |  |  |
| Chr I B | *cyh1* | *leu2* | 670^b^ |  |  |  |  |  |  |  |  | 26 | 0 | 22 | 48 | 23 | 31 |
|  | *leu2* | *ade4* | 924 |  |  |  |  |  |  |  |  | 30 | 0 | 18 | 48 | 19 | 24 |
|  |  |  |  |  |  |  |  |  |  |  |  |  |  |  |  |  |  |
| Chr II A | *ade7* | *his3* | 331 |  | 15 | 8 | 38 | 61 | ND | ND |  | 18 | 0 | 10 | 28 | 18 | 22 |
|  | *his3* | *can1* | 402^b^ |  | 20 | 2 | 39 | 61 | 42 | 61 |  | 21 | 0 | 7 | 28 | 13 | 14 |
|  | *can1* | *leu1* | 84^b^ |  | 31 | 1 | 29 | 61 | 29 | 36 |  | 22 | 1 | 5 | 28 | 20 | 14 |
|  |  |  |  |  |  |  |  |  |  |  |  |  |  |  |  |  |  |
| Chr III A | *ura4* | *ade6* | 1201 |  | 12 | 10 | 41 | 63 | ND | ND |  | 34 | 1 | 19 | 54 | 23 | 25 |
|  | *ade6* | *arg1* | 297 |  | 16 | 6 | 41 | 63 | 61 | ND |  | 41 | 0 | 13 | 54 | 12 | 14 |
|  |  |  |  |  |  |  |  |  |  |  |  |  |  |  |  |  |  |
| Chr III B | *arg1* | *ade5* | 799 |  | 7 | 11 | 38 | 56 | ND | ND |  | 14 | 2 | 25 | 41 | 45 | 61 |

PD = parental ditype; NPD = non-parental ditype; T = tetratype. Our criterion for linkage in tetrad analysis is PD > NPD, with statistical significance p < 0.05 by chi-squared test. A more stringent criterion would be p < 0.01 (Kohli et al. 1977).

a: Map distance d_p_ is based on Perkins' formula, and map distance d_l_ is derived from maximum likelihood estimate (see Methods), as distances based on Perkins’ formula are likely to be underestimates when NPD > 5% of total tetrads. ND = not determined, because data do not suggest linkage.

b: Estimated distance; see Table 2.
