# Supplementary material for: Improved tools for efficient mapping of fission yeast genes: identification of microtubule nucleation modifier mod22-1 as an allele of chromatin- remodelling factor gene swr1
Source: Yeast. 2008 Dec;25(12):913–25. doi: 10.1002/yea.1639 (PMC2964509; doi:10.1002/yea.1639)
Supplement: Supplementary file 2 [file yea0025-0913-SD2.doc]

**Table S2. Distances between markers, from random spore analysis**

| Mapping strain | *swi5* background | Marker  (A) | Marker  (B) | Physical distance  (A to B; kb) | % recombinants | N | Chi^2^ | p |
| --- | --- | --- | --- | --- | --- | --- | --- | --- |
| Chr I B | *swi5+* | *cyh1* | *leu2* | 670^a^ | 53 | 120 | 0.3 | 0.583 |
|  |  | *leu2* | *ade4* | 924 | 51 | 120 | 0.033 | 0.855 |
|  |  |  |  |  |  |  |  |  |
| Chr II B | *swi5+* | *leu3* | *lys4* | 818 | 44 | 280 | 4.629 | 0.03 |
|  |  | *lys4* | *arg5* | 589 | 45 | 280 | 3.214 | 0.07 |
|  |  |  |  |  |  |  |  |  |
| Chr II B | *swi5∆* | *leu3* | *lys4* | 818 | 7.4 | 560 | 408 | <0.0001 |
|  |  | *lys4* | *arg5* | 589 | 8.7 | 560 | 381 | <0.0001 |
|  |  |  |  |  |  |  |  |  |

a: Estimated distance; see Table 2.
