# Supplementary material for: Improved tools for efficient mapping of fission yeast genes: identification of microtubule nucleation modifier mod22-1 as an allele of chromatin- remodelling factor gene swr1
Source: Yeast. 2008 Dec;25(12):913–25. doi: 10.1002/yea.1639 (PMC2964509; doi:10.1002/yea.1639)
Supplement: Supplementary file 3 [file yea0025-0913-SD3.doc]

**Table S3. Lists of up- and downregulated genes common to *mod22-1*, *swr1∆* and *pht1∆* mutants. ('RC' indicates reverse-complement)**

**Genes down >1.25X in all mutants:**

| **Primer name** | **Common name** | **Systematic name** | **Annotation** |
| --- | --- | --- | --- |
| pht1: pi001.RC |  |  |  |
| C1259.07 |  | SPCC1259.07 | chromatin silencing protein, |
| C4D7.10c |  | SPAC4D7.10c | histone acetyltransferase SAGA complex subunit, |
| c9E9.17c |  | SPAC9E9.17c | sequence orphan, |
| C1778.09.RC |  |  |  |
| C21.03c |  | SPBC21.03c | hypothetical protein (broad species distribution), |
| C1223.13 |  | SPCC1223.13 | transcription factor, |
| C23G7.10c |  | SPBC23G7.10c | NADH-dependent flavin oxidoreductase, |
| C1F12.03c |  | SPAC1F12.03c | sequence orphan, |
| cut12: stf1 | stf1;cut12 | SPBC649.05 | spindle pole body protein, cut12, stf1 |
| spc24 | efc25 | SPBC336.03 | exchange factor Cdc25p-like, efc25 |
| C16E9.03c |  | SPBC16E9.03c | conserved fungal protein, |
| C29B12.10c |  | SPAC29B12.10c | OPT oligopeptide transporter family, |
| P26C9.03c |  | SPBP26C9.03c | iron ion transporter, |
| rng2 | rng2 | SPAC4F8.13c | IQGAP, rng2 |
| PB2B2.18 |  | SPBPB2B2.18 | dubious, |
| C13F5.04c |  | SPAC13F5.04c | sequence orphan, |
| prl12 |  | SPNCRNA.12 |  |
| C36.01c |  | SPBC36.01c | spermidine family transporter, |
| C887.21c |  | SPBC1105.18c | peptidyl tRNA hydrolase |
| C25H2.03 |  | SPBC25H2.03 | HEAT repeat, |
| C1093.01 |  | SPAC1093.01 | PPR domains |
| C959.06c |  | SPAC959.06c | sequence orphan, |
| C622.01c |  | SPCC622.01c | sequence orphan, |
| C806.08c |  | SPAC806.08c | sequence orphan, mod21 |
| C1861.03 | mak10 | SPBC1861.03 | NatC N-acetyltransferase complex subunit, mak10 |
| C1198.01 |  | SPBC1198.01 | glutathione-dependent formaldehyde dehydrogenase, |
| C2D10.17 | clr1 | SPBC2D10.17 | cryptic loci regulato, clr1 |
| C1861.04c |  | SPBC1861.04c | RNA-binding protein, |
| C1393.08.RC |  |  |  |
| C2F12.03c.RC |  |  |  |
| C613.12c | dos1;cmc1;raf1;clr8 | SPCC613.12c | Rik1-associated factor |
| PYUG7.06 | hag1 | SPAPYUG7.06 | PPPDE peptidase family, |
| CUNK4.09 |  | SPACUNK4.09 | conserved protein, |
| C23A1.02c |  | SPAC23A1.02c | calcineurin-like phosphoesterase, |
| SNRNA.01 |  | SPSNRNA.01 |  |
| C18.12c |  | SPCC18.12c | DUF652, |
| C806.10 |  | SPAC24B11.14 | sequence orphan |
| ypt4 | ypt4 | SPAC1B3.11c | GTPase, ypt4 |
| C17G8.02 |  | SPAC17G8.02 | uridine ribohydrolase, |
| RRNA.02 |  | SPRRNA.02 |  |
| pck2: sts6: pkc1 | pkc1;pck2;sts6 | SPBC12D12.04c | protein kinase C (PKC)-like Pck2 |
| C17G8.12 |  | SPAC17G8.12 | sequence orphan, |
| meu11RC |  | SPNCRNA.17 |  |
| C70.09c |  | SPCC70.09c | conserved fungal protein, |
| C1827.03c |  | SPCC1827.03c | acetyl-CoA ligase, |
| C1672.09 |  | SPCC1672.09 | triglyceride lipase-cholesterol esterase, |
| C959.05c |  | SPAC959.05c | protein disulfide isomerase, |
| C23C4.14 | alg1 | SPAC23C4.14 | mannosyltransferase complex subunit Alg1 |
| C4A8.10 |  | SPAC4A8.10 | lipase, |
| prl60 |  | SPNCRNA.60 |  |
| RRNA.32 |  | SPRRNA.32 |  |
| C12B10.10 |  | SPAC12B10.10 | sequence orphan, |
| C8C9.01 | fhl1 | SPAC1142.08 | fork head transcription factor Fhl1 |
| C1795.13 |  | SPCC1742.01 | glycoprotein |
| C825.04c |  | SPCC825.04c | N-acetyltransferase, |
| C3D6.10 | eth1;apn2 | SPBC3D6.10 | AP-endonuclease Apn2 |
| C20F10.02c |  | SPBC20F10.02c | conserved eukaryotic protein, |
| misc_RNA_2.2.51.RC |  | SPNCRNA.92 |  |
| C354.08c |  | SPBC354.08c | DUF221, |
| NCRNA.76 |  | SPNCRNA.76 |  |
| csn5 | csn5 | SPAC1687.13c | COP9/signalosome complex subunit Csn5 |
| C1685.04 |  | SPBC1685.04 | sequence orphan, |
| C28F2.05c |  | SPBC28F2.05c | aldo/keto reductase, |
| C126.04c |  | SPCC126.04c | SAGA associated factor, |
| C24H6.11c |  | SPAC24H6.11c | sulfate transporter, |
| C140.05 | ppk1 | SPAC110.01 | serine/threonine protein kinase, ppk1 |
| rRNA_2.2.16 |  |  |  |
| C1919.07 |  | SPCC1919.07 | sequence orphan, |
| misc_RNA_4.4.31.RC |  |  |  |
| C16A3.19 |  | SPBC16A3.19 | histone acetyltransferase complex subunit, |
| C553.10 |  | SPCC553.10 | glycoprotein, |
| NCRNA.113 |  | SPNCRNA.113 |  |
| alp6 | alp6 | SPBC428.20c | gamma tubulin complex subunit Alp6 |
| C3H8.01 |  | SPAC13A11.06 | pyruvate decarboxylase |
| C14F5.01 |  | SPBC14F5.01 | sequence orphan |
| P21534 | cox2 | SPMIT.11 | cytochrome c oxidase 2, cox2 |
| C5H10.09c |  | SPAC5H10.09c | 3-methyl-2-oxobutanoate hydroxymethyltransferase, |
| C947.04 |  | SPBC947.04 | glycoprotein, |
| CPT2R1.07c |  | SPBCPT2R1.07c |  |
| C227.06 |  | SPAC227.06 | Rab GTPase binding, |
| C56F8.06c | alg10 | SPAC56F8.06c | dolichyl-phosphate-glucose-glycolipid alpha-glucosyltransferase Alg10 |
| C3G9.02 |  | SPAC3G9.02 | 3-oxoacyl-[acyl-carrier protein] reductase Oar2 |
| C56F8.12.RC |  |  |  |
| SNORNA.50 |  | SPSNORNA.50 |  |
| C106.17c |  | SPBC106.17c | homoserine O-acetyltransferase, |
| rpl41-2 | rpl4102;rpl41;rpl41-2;rpl41-2B.01c | SPAC3F10.18c | 60S ribosomal protein L41, rpl4102, rpl41-2, rpl41 |
| gpa2: git8 | gpa2;git8 | SPAC23H3.13c | heterotrimeric G protein alpha-2 subunit Gpa2 |
| C576.13 | swc5 | SPCC576.13 | chromatin remodeling complex subunit Swc5 |
| C12B10.04 |  | SPAC12B10.04 | tubulin-tyrosine ligase, |
| RRNA.37 |  | SPRRNA.37 |  |
| C57A7.05 |  | SPAC57A7.05 | conserved protein, |
| C3H1.11 |  | SPAC3H1.11 | transcription factor, hsr1 |
| P22191 | cox1I1 | SPMIT.02 | DNA binding endonuclease, |
| C13A11.03 | mug32;mcp7 | SPAC13A11.03 | meiosis specific coiled-coil protein Mcp7 |
| C212.09c |  | SPAC212.09c |  |
| C688.02c | mis14 | SPAC688.02c | kinetochore protein Mis14 |
| P21547 | urfA | SPMIT.08 | mitochondrial ribosomal small subunit, |
| alp41 | alp41 | SPAC22F3.05c | ADP-ribosylation factor, alp41 |
| C977.15 |  | SPAC977.15 | dienelactone hydrolase family, |
| C56F2.13 | cnp3 | SPBC1861.01c | CENP-C, cnp3 |
| PB24D3.07c |  | SPAPB24D3.07c | sequence orphan, |
| P14575 | cox3 | SPMIT.04 | cytochrome c oxidase 3, cox3 |
| P21535 | atp6 | SPMIT.07 | F0-ATPase subunit 6, atp6 |
| rds1 | rds1 | SPAC343.12 | conserved fungal protein, rds1 |
| P21537 | atp9 | SPMIT.10 | F0-ATPase subunit 9, atp9 |
| C29A3.13 |  | SPBC29A3.13 | PWWP domain protein, |
| RRNA.01 |  | SPRRNA.01 |  |
| abp2 | abp2 | SPBC1861.02 | ARS binding protein Abp2 |
| P21536 | atp8 | SPMIT.09 | F0-ATPase subunit 8, atp8 |
| RRNA.08 |  | SPRRNA.08 |  |
| P07657 | cox1 | SPMIT.01 | cytochrome c oxidase 1, cox1 |
| misc_RNA_4.4.31 |  | SPNCRNA.98 |  |
| P05501 | cob | SPMIT.05 | cytochrome b, cob |
| C1348.13 |  | SPBC1348.13 |  |
| P05511 | cobI | SPMIT.06 | DNA binding endonuclease, |
| C11C11.12 |  | SPBC11C11.12 |  |
| P22190 |  | SPMIT.03 | DNA binding endonuclease, |
| C186.05c |  | SPAC186.05c | hypothetical protein, |
| PB8B6.03.RC |  |  |  |
| C132.04c |  | SPCC132.04c | NAD dependent glutamate dehydrogenase, |

**Genes down >1.5X in all mutants:**

| **Primer name** | | **Common name** | **Systematic name** | **Annotation** |
| --- | --- | --- | --- | --- |
| C947.04 | |  | SPBC947.04 | glycoprotein, |
| C3G9.02 | |  | SPAC3G9.02 | 3-oxoacyl-[acyl-carrier protein] reductase Oar2 |
| C56F8.12.RC | |  |  |  |
| SNORNA.50 | |  | SPSNORNA.50 |  |
| gpa2: git8 | | gpa2;git8 | SPAC23H3.13c | heterotrimeric G protein alpha-2 subunit Gpa2 |
| C576.13 | | swc5 | SPCC576.13 | chromatin remodeling complex subunit Swc5 |
| C12B10.04 | |  | SPAC12B10.04 | tubulin-tyrosine ligase, |
| C57A7.05 | |  | SPAC57A7.05 | conserved protein, |
| C3H1.11 | |  | SPAC3H1.11 | transcription factor, hsr1 |
| P21547 | | urfA | SPMIT.08 | mitochondrial ribosomal small subunit, |
| alp41 | | alp41 | SPAC22F3.05c | ADP-ribosylation factor, alp41 |
| C56F2.13 | | cnp3 | SPBC1861.01c | CENP-C, cnp3 |
| PB24D3.07c | |  | SPAPB24D3.07c | sequence orphan, |
| P14575 | | cox3 | SPMIT.04 | cytochrome c oxidase 3, cox3 |
| P21535 | | atp6 | SPMIT.07 | F0-ATPase subunit 6, atp6 |
| rds1 | | rds1 | SPAC343.12 | conserved fungal protein, rds1 |
| P21537 | | atp9 | SPMIT.10 | F0-ATPase subunit 9, atp9 |
| C29A3.13 | |  | SPBC29A3.13 | PWWP domain protein, |
| RRNA.01 | |  | SPRRNA.01 | |
| abp2 | | abp2 | SPBC1861.02 | ARS binding protein, abp2 |
| P21536 | | atp8 | SPMIT.09 | F0-ATPase subunit 8, atp8 |
| RRNA.08 | |  | SPRRNA.08 | |
| P07657 | | cox1 | SPMIT.01 | cytochrome c oxidase 1, cox1 |
| misc_RNA_4.4.31 |  | | SPNCRNA.98 | |
| P05501 | | cob | SPMIT.05 | cytochrome b, cob |
| C1348.13 | |  | SPBC1348.13 | |
| P05511 | | cobI | SPMIT.06 | DNA binding endonuclease, |
| C11C11.12 | |  | SPBC11C11.12 | |
| P22190 | |  | SPMIT.03 | DNA binding endonuclease, |
| PB8B6.03.RC | | |  |  |

**Genes down >2X in all mutants:**

| **Primer name** | **Common name** | **Systematic name** | **Annotation** |
| --- | --- | --- | --- |
| C56F2.13 | cnp3 | SPBC1861.01c | CENP-C, cnp3, SPBC56F2.13 |
| P21535 | atp6 | SPMIT.07 | F0-ATPase subunit 6, atp6 |
| P21537 | atp9 | SPMIT.10 | F0-ATPase subunit 9, atp9 |
| C29A3.13 |  | SPBC29A3.13 | PWWP domain protein, |
| RRNA.01 |  | SPRRNA.01 |  |
| abp2 | abp2 | SPBC1861.02 | ARS binding protein, Abp2 |
| P21536 | atp8 | SPMIT.09 | F0-ATPase subunit 8, atp8 |
| P05501 | cob | SPMIT.05 | cytochrome b, cob |
| C1348.13 |  | SPBC1348.13 |  |
| P05511 | cobI | SPMIT.06 | DNA binding endonuclease, |
| C11C11.12 |  | SPBC11C11.12 |  |
| P22190 |  | SPMIT.03 | DNA binding endonuclease, |
| PB8B6.03.RC | |  |  |

**Genes up >1.25X in all mutants:**

| **Primer name** | **Common name** | **Systematic name** | **Annotation** |
| --- | --- | --- | --- |
| C1002.19 |  | SPAC1002.19 | GTP cyclohydrolase, urg1 |
| C1002.17c |  | SPAC1002.17c | uracil phosphoribosyltransferase, |
| isp5 | isp5 | SPAC1039.09 | amino acid permease family, isp5 |
| hsp16 | hsp16 | SPBC3E7.02c | heat shock protein, hsp16 |
| C23D3.12 |  | SPAC23D3.12 | inorganic phosphate transporter |
| C660.05 |  | SPBC660.05 | hypothetical protein |
| prl20 |  | SPNCRNA.20 |  |
| C8E4.01c |  | SPBC8E4.01c | inorganic phosphate transporter |
| C212.11 |  | SPAC212.11 | RecQ type DNA helicase, tlh1 |
| C215.10 |  | SPBC215.10 | haloacid dehalogenase-like hydrolase |
| ght5 | ght5 | SPCC1235.14 | hexose transporter, ght5 |
| C11D3.04c |  | SPAC11D3.04c | SnoaL |
| ubc15 | ubcX;ubc15 | SPBC1105.09 | ubiquitin conjugating enzyme, ubc15 |
| C5H10.10 |  | SPAC5H10.10 | NADPH dehydrogenase |
| C212.06c |  | SPAC212.06c | |
| C9.08c |  | SPAC9.08c | steroid reductase, |
| b8647-5 |  | SPCC737.04 | UPF0300 family, |
| C359.06 |  | SPBC359.06 | adducin N-terminal domain protein, |
| C21C3.09c |  | SPBC21C3.09c | fumarylacetoacetate (FAA) hydrolase, |
| C790.03 |  | SPCC790.03 | rhomboid family protease, |
| C548.06c |  | SPCC548.06c | hexose transporter, ght8 |
| pB18E9.05c |  | SPAPB18E9.05c | dubious, |
| pB21E7.04c |  | SPBPB21E7.04c | S-adenosylmethionine-dependent methyltransferase |
| wis2 | cyp5;wis2 | SPAC1B3.03c | cyclophilin, wis2 |
| C16D10.08c |  | SPBC16D10.08c | heat shock protein, |
| wtf24 | wtf24 | SPCC830.02 |  |
| C713.11c |  | SPBC713.11c | UPF0057 family, pmp3 |
| C1705.02 |  | SPAC1705.02 | conserved eukaryotic protein, |
| C1711.08 |  | SPBC1711.08 | chaperone activator, |
| PB16A4.06c |  | SPCPB16A4.06c | dubious, |

**Genes up >1.5X in all mutants:**

| **Primer name** | **Common name** | **Systematic name** | **Annotation** |
| --- | --- | --- | --- |
| C1002.19 |  | SPAC1002.19 | GTP cyclohydrolase, urg1 |
| isp5 | isp5 | SPAC1039.09 | amino acid permease family, isp5 |
| C23D3.12 |  | SPAC23D3.12 | inorganic phosphate transporter, |
| C8E4.01c |  | SPBC8E4.01c | inorganic phosphate transporter |
| C212.11 |  | SPAC212.11 | RecQ type DNA helicase, tlh1 |
| C11D3.04c |  | SPAC11D3.04c | SnoaL, |
